# Human embryonic stem cells display a pronounced sensitivity to the cyclin dependent kinase inhibitor Roscovitine

**DOI:** 10.1186/s12860-019-0222-3

**Published:** 2019-08-28

**Authors:** Guillermo A. Videla-Richardson, Verónica A. Furmento, Carolina P. Garcia, Olivia Morris-Hanon, Gustavo E. Sevlever, Leonardo Romorini, María E. Scassa

**Affiliations:** Laboratorios de Investigación Aplicada en Neurociencias (LIAN-CONICET), Fundación para la Lucha contra las Enfermedades Neurológicas de la Infancia (FLENI), Belén de Escobar, Provincia de Buenos Aires Argentina

**Keywords:** Human embryonic stem cells, Roscovitine, Cyclin-dependent kinases, Apoptosis

## Abstract

**Background:**

The essentially unlimited expansion potential and the pluripotency of human embryonic stem cells (hESCs) make them attractive for cell-based therapeutic purposes. Although hESCs can indefinitely proliferate in culture, unlike transformed cancer cells, they are endowed with a cell-intrinsic property termed mitochondrial priming that renders them highly sensitive to apoptotic stimuli. Thus, all attempts to broaden the insights into hESCs apoptosis may be helpful for establishing pro-survival strategies valuable for its in vitro culture and further use in clinical applications. Cyclin-dependent kinases (CDKs), a family of serine/threonine protein kinases originally identified as regulators of the eukaryotic cell cycle, can also regulate transcription and differentiation. Moreover, there are compelling data suggesting that its activities are involved in certain apoptotic programs in different cell types. Currently, it is not completely determined whether CDKs regulate apoptotic processes in rapidly proliferating and apoptosis-prone hESCs. In this study, to elucidate the effect of CDKs inhibition in hESCs we used Roscovitine (ROSC), a purine analogue that selectively inhibits the activities of these kinases.

**Results:**

Inhibition of CDKs by ROSC triggers programmed cell death in hESCs but not in proliferating somatic cells (human fibroblasts). The apoptotic process encompasses caspase-9 and -3 activation followed by PARP cleavage. ROSC treatment also leads to p53 stabilization, which coincides with site-specific phosphorylation at serine 46 and decreased levels of Mdm2. Additionally, we observed a transcriptional induction of *p53AIP1*, a repression of pro-survival factor Mcl-1 and an up-regulation of pro-apoptotic BH3-only proteins NOXA and PUMA. Importantly, we found that the role of CDK2 inhibition appears to be at best accessory as an active CDK2 is not required to ensure hESCs survival.

**Conclusion:**

Our experimental data reveal that hESCs, contrary to fibroblasts, exhibit a pronounced sensitivity to ROSC.

## Background

Human embryonic stem cells (hESCs) are pluripotent cells exceptionally dedicated to rapid unlimited proliferation [1]. At the pre-implantation stage of the blastocyst, as well as in vitro culture, hESCs repeatedly traverse the cell cycle and undergo successive symmetrical divisions to give rise to an equivalent progeny that exhibit identical self-renewal and differentiation properties. In comparison to proliferating somatic cells, hESCs show atypical cell cycle properties such as short doubling time (15–16 h), truncated G1 phase (3–4 h) and reduced or absent checkpoints [2, 3]. Although hESCs can indefinitely proliferate in culture they are endowed with a cell-intrinsic property termed mitochondrial priming that renders them highly sensitive to apoptotic stimuli [4].

Cyclin-dependent kinases (CDKs), a family of serine/threonine protein kinases originally identified as regulators of the eukaryotic cell cycle, can also regulate transcription and, in certain cell types, differentiation [5, 6]. Until present, 20 CDKs have been characterized and have been separated into two subfamilies: cell-cycle-related subfamilies (CDK1, CDK4 and CDK5) and transcription-associated subfamilies (CDK7, CDK8, CDK9, CDK11 and CDK20) [6]. The catalytic activity of CDKs essentially depends on their binding to regulatory subunits [7, 8]. CDKs activity is also modulated by CDK kinases, that include the CDK activating complex CAK (comprising CDK7, cyclin H and Mat1), by Wee and Myt1 kinases, by CDK phosphatases (CDC25 phosphatases), and by CDK inhibitors (CKIs), including the INK4 (p15^INK4b^, p16^INK4a^, p18^INK4c^, and p19^INK4d^) and the Cip/Kip (p21^Cip1^, p27^Kip1^, and p57^Kip2^) families [5, 7]. Interestingly, it has been shown that hESCs display high levels of CDK activity in part due to the absence or very weak expression of CKIs [9, 10].

CDK inhibitors, representing a well-defined group of biologically active compounds, are structurally related to adenosine-5′-triphosphate (ATP). The 2,6,9-tri-substituted purine chemistry defines a family of drugs, such as Olomoucine, Roscovitine (ROSC) and Purvalanol, that selectively inhibit the activities of CDKs by specific binding to their ATP-binding pocket [11]. ROSC is a broad-range purine inhibitor, which effectively inhibits CDK1, CDK2, CDK5, CDK7 and CDK9, but is a poor inhibitor for CDK4 and CDK6 [12, 13]. In proliferating somatic cells, ROSC arrests the cell cycle in G1, S, or in G2/M phases, depending on the cell type used, the concentration and the time of exposure. Although the primary reason for this arrest is the acute inhibition of CDK1 and CDK2 [14], there are several indirect mechanisms by which ROSC can disturb cell cycle progression. The immense majority of these indirect mechanisms arises from changes in gene expression profiles in ROSC-treated cells. It is now known that ROSC decreases transcription by inhibiting CDK7 and CDK9, which are responsible for the phosphorylation of the carboxy-terminal domain (CTD) of the RNA polymerase II largest subunit, an activity that results relevant for transcription initiation and elongation [15].

Furthermore, in some cellular contexts CDK inhibitors can induce programmed cell death [16–18]. In this regard, ROSC appears to have contradictory effects towards apoptosis depending on the cycling status of the cell. In highly dividing cells ROSC stimulates apoptosis; conversely, in non-dividing or differentiating cells this CDK inhibitor exerts a protective effect [19, 20]. Importantly, to date it has not been fully addressed whether CDKs activities are universally required in apoptotic processes. Nevertheless, despite the absence of a model of CDKs involvement in programmed cell death, there are convincing data that suggest that its activities are required in a subset of apoptosis programs in certain cell types [21]. To this end, potential mechanisms related to the regulation of apoptosis by CDKs that include interactions between the CDKs and the p53 signaling pathways have been described [22, 23]. It thus appear that CDKs have cell-type specific functions and that compensatory roles exist among different CDK family members, which could play important roles in the control of proliferation and apoptosis.

The strict control of cell proliferation and cell death is crucial for embryonic development. Many of the gene products which appear to control apoptotic tendencies are cell cycle regulators; thus, the control of cell death and the one of cell cycle seem to be closely related processes. In this sense, whether CDKs regulate apoptotic processes in rapidly proliferating and apoptosis-prone hESCs remain to be determined, and if so, the underlying mechanisms involved await elucidation. In the present study we found that in hESCs, inhibition of CDKs by ROSC induces cell cycle arrest and apoptosis. The apoptotic effect of ROSC is manifested by the activation of caspase-9 and caspase-3. This CDK inhibitor also led to stabilization and site-specific phosphorylation of p53 at serine 46 concomitantly with down-regulation of murine double minute 2 (*mdm2*) mRNA and protein levels. Moreover, ROSC treatment induces changes in the expression levels of pro-survival Bcl-2 protein, myeloid cell leukemia-1 (Mcl-1) and its BH3-only binding partners: p53-upregulated modulator of apoptosis (PUMA) and phorbol-12-myristate-13-acetate-induced protein 1 (PMAIP1/NOXA) shifting the balance between anti- and pro-apoptotic factors towards cell death. Notably, impairment of CDK2 activity was not a critical event in ROSC-induced apoptosis. Therefore, using small molecule CDK inhibitors we aimed to shed light on the cellular mechanisms that dictate hESCs fate decisions.

## Results

### Onset of cell cycle arrest in hESCs exposed to ROSC

**Fig. 1:**
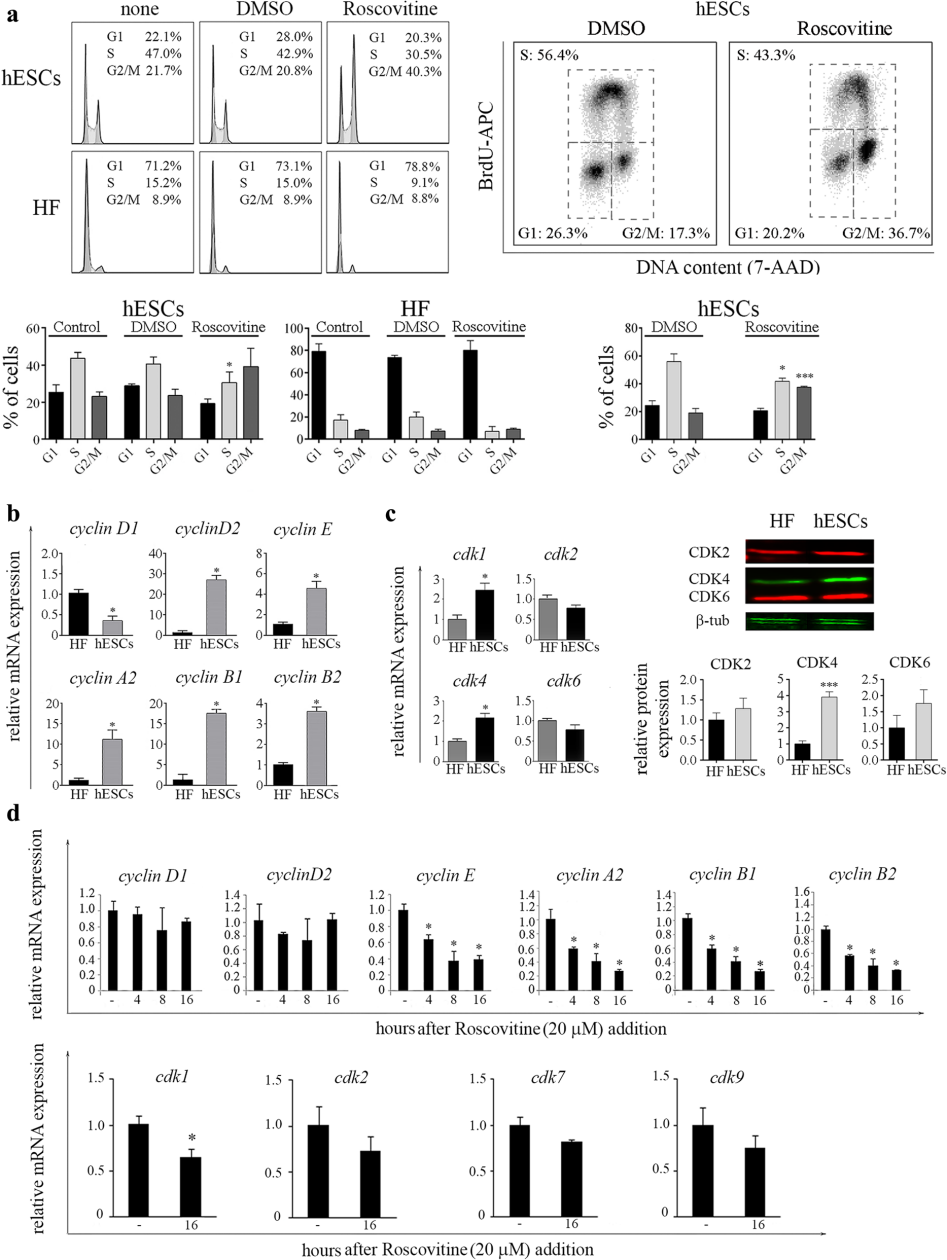
ROSC affects cell cycle distribution and key cell cycle regulators expression. **a** hESCs were exposed to ROSC or DMSO for 16 h. Cell cycle distribution of asynchronously growing or ROSC-treated H9 and HF was determined by flow cytometry analysis of cellular DNA content following cell staining with PI (left panel). Cells were pulse labeled with BrdU for 30 min prior to harvesting and stained with anti-BrdU-APC conjugate and with7-amino-actinomycin D (7-AAD) for determination of DNA synthesis and DNA content respectively (right panel). Bar graphs summarizing flow cytometry cell cycle profile analysis. Error bars represent means ± SD from three independent experiments (lower panels). **b** Comparison of mRNA expression levels *for cyclin D1, D2, E, A2, B1* and *B2* in hESCs and HF assessed by Real Time RT-PCR (left panel). *Rpl7*expression served as normalizer. Graph shows mRNA fold change relative to HF. The mean ± SEM from three independent experiments are shown. **c** Comparison of mRNA expression levels of *cdk1, cdk2, cdk4* and *cdk6* in hESCs and HF analyzed by Real Time RT-PCR (left panel). Representative Western blot images of CDK2, CDK4 and CDK6 (right panel). β-Tubulin served as loading control. Bar graphs show densitometric quantification. Data are expressed as means ± SD (left panel). **d** Time course analysis of mRNA levels of *cyclin D1, D2, E, A2, B1, B2* and *cdk1, cdk2, cdk7* and *cdk9* were assessed by Real Time RT-PCR in ROSC-treated or untreated hESCs. *Rpl7* expression served as normalizer. Graph shows mRNA fold change relative to untreated cells. The mean ± SEM from three independent experiments are shown. In all cases paired Student’s *t* test was used to test for significant differences *P < 0.05, ***P < 0.001

As previously mentioned, ROSC has the potential to perturb cell cycle progression in G1, S or G2/M depending on the dose and time of exposure in various cell types, ranging from numerous cancer cell lines to keratinocytes and fibroblasts [24, 25]. Thus, to evaluate whether ROSC has the potential to promote cell cycle arrest in asynchronously growing hESCs, we determined the percentage of cells in each phase of the cell cycle after a 16 h exposure period by quantification of DNA content using PI staining. Flow cytometric analysis revealed that ROSC-treated cells retained a significantly higher proportion of cells in G2/M (40%) than those treated with DMSO (21%) (Fig. 1) a. Alternatively, DNA synthesis was analyzed by a biparametric flow cytometry analysis of BrdU incorporation vs. DNA content. As seen in Fig. 1 a BrdU incorporation assays clearly show a marked increase in G2/M population after ROSC treatment (ROSC-treated 37% vs. DMSO-treated 17%). This CDK inhibitor also caused a decrease in the percentage of cells that were actively replicating DNA (S phase). By contrast, when HF were exposed to ROSC we observed a slight increase in G1 population with a concomitant decrease in S population (Fig. 1a).

Next, we aimed to compare the mRNA expression levels of key cell cycle regulators in hESCs and HF. To address this issue, we measured different cyclin mRNAs in both cell types by real time RT-PCR. Consistent with previous reports, we determined that *cyclin D2* mRNA is the predominant D-type cyclin gene expressed in hESCs (H9) (data not shown) [26]. Additionally, we observed that asynchronously growing hESCs express higher levels of *cyclin D2, E, A2, B1* and *B2* mRNAs than HF (Fig. 1b). Then, we analyzed the expression levels of CDK1, CDK2, CDK4 and CDK6 in pluripotent cells and HF. We found that H9 cells express significantly higher levels of *cdk*1 and *cdk*4 mRNAs than HF (Fig. 1c, left panel). We also evaluated the abundance of the major CDKs that are activated during interphase (CDK2, CDK4 and CDK6) by Western blot (Fig. 1c, right panel). We found a good correlation between them mRNA levels and the corresponding protein products in each cell type.

We then tested whether ROSC affects the expression levels of key cell cycle regulators. To do so, we analyzed *cyclin D1, D2, E, A2, B1* and *B2* mRNAs expression at different time points after ROSC addition (20 μM). We determined that almost all cyclins mRNA expression levels were reduced as soon as 4 h post-treatment respect to those exhibited by DMSO-treated control cells, except for *cyclin D1* and *D2*, whose levels remained fairly constant over the time frame the experiments were conducted. The observation that the transcript levels of essential regulators of G2/M transition *(cyclin A2, B1* and *B2)* were robustly down-regulated may provide a possible mechanism by which ROSC can cause cell cycle arrest in G2/M phase in pluripotent cells.

Concerning to cell cycle regulation, it has been reported that a pure R-enantiomer of ROSC, CYC202, decreases the expression of several transcripts involved directly or indirectly in cell cycle progression such as CDK1, CDK7 and CDK9, among others [27]. Thus, to further explore whether ROSC has also the potential to affect the expression levels of these genes in pluripotent cells we performed real time RT-PCR analysis. We found that *cdk1* transcript was slightly although significantly down-regulated in hESCs, while *cdk*2, *cdk*7 and *cdk*9 mRNA levels remained unaffected when challenged with ROSC for 16 h (Fig. 1d, bottom panel).

### ROSC triggers apoptosis of hESCs

**Fig. 2:**
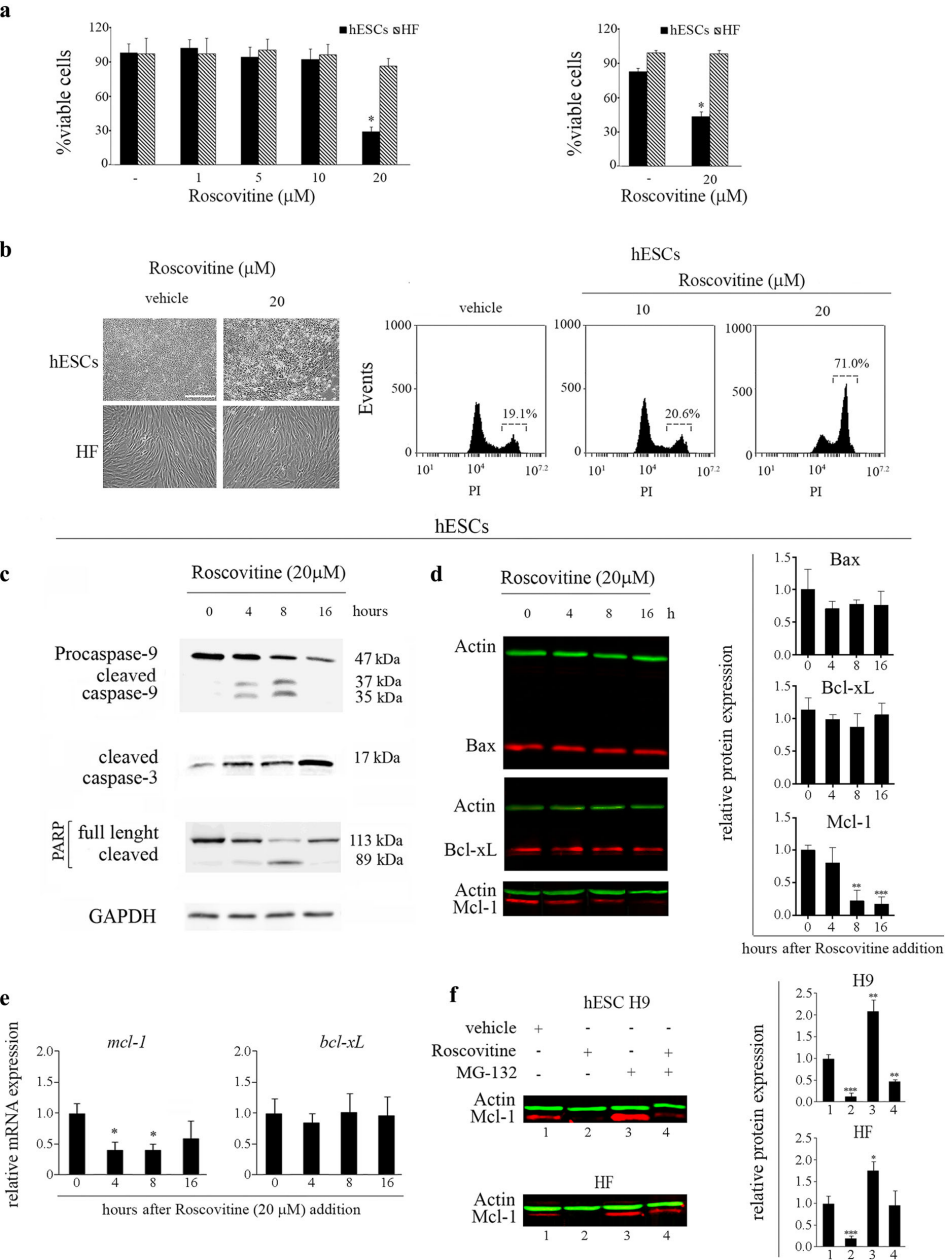
ROSC induces apoptosis in H9 hESC line. **a** H9 cells and HF were treated with increasing concentrations of ROSC (0–20 μM) over a 16 h period. Cell viability was measured by the XTT/PMS vital dye assay (left panel) and by Trypan blue exclusion method (right panel). Bar graphs show the percentage of viable cells. Each bar represents the mean ± SEM of three independent experiments performed in quadruplicates. **b** Representative photomicrographs of H9 colonies and HF treated or not with 20 μM ROSC over a 16 h period. The scale bar represents 100 μm (right panel). Representative histograms of PI stained cells exposed or not to ROSC for 16 h. Percentage of PI+ cells was determined by flow cytometric analysis (left panel). **c** Time course of caspase-9 and caspase-3 activation and PARP cleavage in hESCs upon ROSC treatment was analyzed by Western blotting with anti-caspase-9, anti-active caspase-3 and anti-PARP specific antibodies. GAPDH served as loading control. **d** Representative Western blot in H9 cells of Bax, Bcl-xL and Mcl-1 at different time points after ROSC addition. Bar graphs show densitometric quantification. Data are expressed as means ± SD (left panel). **e** A time-course analyses of *mcl-1* and *bcl-xL* mRNA expression levels by Real Time RT-PCR in ROSC-treated or untreated hESCs. *Rpl7* expression served as normalizer. Graph shows mRNA fold change relative to untreated cells. Each bar represents the mean ± SEM of three independent experiments. **f** H9 cells and HF were incubated in the absence or presence of ROSC (20 μM) or MG-132 (5 μM) alone or combined. Mcl-1 level of expression was verified by immunoblotting. Actin served as loading control. Bar graphs show densitometric quantification. A paired Student’s t test was used to compare ROSC-treated samples to untreated controls *P < 0.05, **P < 0.01, ***P < 0.001

Next, we evaluated the possible effects of ROSC on hESCs and HF viability. To address this issue, we determined the number of viable cells after continuous exposure to ROSC for 16 h at concentrations ranging from 0 to 20 μM using a XTT/PMS vital dye assay. As shown in Fig. 2a no evident effects were observed with ROSC at 1 to 10 μM, but cell viability was strongly reduced in the presence of ROSC at 20 μM. The percentage of surviving cells decreased to approximately 40% after 16 h of ROSC (20 μM) addition. Moreover, cell death induction by ROSC (20 μM) treatment was further demonstrated by Trypan blue dye-exclusion (Fig. 2a, right panel) and PI staining assays (Fig. 2b, right panel). Conversely, ROSC treatment did not affect the viability of HF at least at the concentrations and periods assayed.

Furthermore, the loss of cell viability was accompanied by changes in cell morphology that included cell detachment and ballooning, both signature features of apoptosis (Fig. 2b, left panel). Hence, to evaluate whether exposure of H9 cells to ROSC led to caspase activation we performed Western blot analysis and found that 4 h after treatment, pro-caspase-9 (47 kDa) was processed generating active fragments (37/35 kDa) (Fig. 2c). Once activated caspase-9 can further cleave and activate downstream effector caspases such as caspase-3. Thus, to further characterize the apoptotic events initiated by ROSC, we used a specific antibody that detects cleaved caspase-3 and determined that this caspase was activated 4 h onwards after treatment (Fig. 2c). Active caspase-3 can cleave downstream substrates involved in apoptotic processes such as poly-(ADP-ribose) polymerase (PARP). Time course experiments revealed the presence of the 89 kDa fragment of PARP 8 h after ROSC addition (Fig. 2c).

### CDK inhibition reduces expression of the anti-apoptotic Bcl-2 family member Mcl-1 in hESCs

The presence of cleaved caspase-9 indicates that ROSC induces apoptosis through activation of the mitochondrial pathway in hESCs. Thus, we assessed the consequences of ROSC treatment on the expression of key mitochondrial-mediated apoptotic players such as Bax, Bcl-xL and Mcl-1. By Western blot analysis we found that there were no substantial changes in Bax or Bcl-xL protein expression levels (Fig. 2d). Conversely, we determined that Mcl-1 levels were markedly reduced in H9 cells at 8 h post treatment and onwards (Fig. 2d). In this regard, we found that in hESCs ROSC also led to a decrease of *mcl-1*mRNA levels without affecting *bcl-xL* transcripts (Fig. 2e). Previous reports have shown that ROSC treatment led to the down-regulation of *mcl*-1 mRNA levels and the loss of Mcl-1 protein product by a mechanism that can be impaired by drugs that inhibit the proteasome [28]. In this regard, we determined that exposure to proteasome inhibitor MG-132 (5 μM) prevented loss of Mcl-1 protein levels in hESCs and HF, even in the presence of ROSC (Fig. 2f).

### ROSC activates p53 pathway in hESCs

**Fig. 3:**
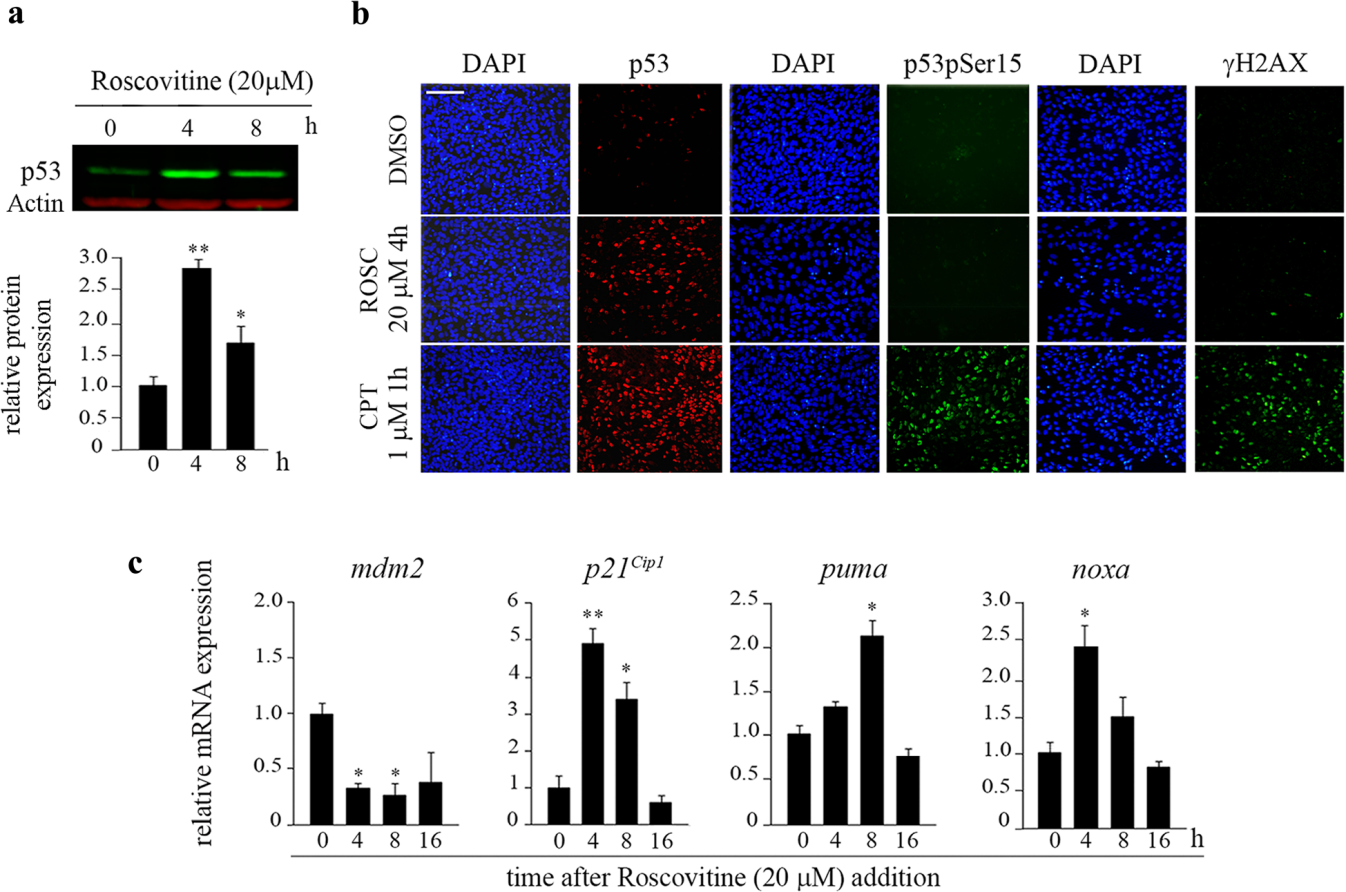
p53 response in ROSC-treated hESCs. **a** Time course of p53 stabilization in hESCs upon ROSC (20 μM) treatment was analyzed by Western blotting with anti-p53 antibody. Actin served as loading control. Bar graph shows densitometric quantification (lower panel). **b** Immunofluorescence staining of untreated, ROSC-treated (20 μM) or Camptothecin-treated (1 μM) H9 cells. Figure shows representative images of hESCs stained with antibodies against γH2AX, p53 and phospho-p53Ser15 (p53pSer15). Nuclei were counterstained with DAPI. The scale bars represent 100 μm. **c** A time-course analyses of p53 targets *mdm2, p21*^*Cip1*^*, puma* and *noxa* mRNA expression levels by Real Time RT-PCR in ROSC-treated or untreated hESCs. *Rpl7* expression was used as normalizer

p53 is a key molecule involved in the regulation of cell cycle progression and the induction of apoptosis in numerous cell types. The activity of this protein is controlled at multiple levels, including protein stability and subcellular localization. Interestingly, it has been reported that substituted purines such as ROSC, mimicking the ATP molecule, activate p53 protein [29]. The above consideration prompted us to explore whether ROSC induces changes in p53 protein level in H9 cells. To address this issue, ROSC-treated (20 μM) hESCs were harvested at different time points and subjected to Western blot assays. As shown in Fig. 3a, enhanced levels of p53 were seen following a 4 h treatment with ROSC and onwards. In parallel, we assessed subcellular localization of p53 after ROSC treatment. Immunofluorescent staining revealed a diffuse pattern of p53-immunoreactivity in untreated hESCs and a clear nuclear localization in ROSC-treated counterparts (Fig. 3b).

**Fig. 4:**
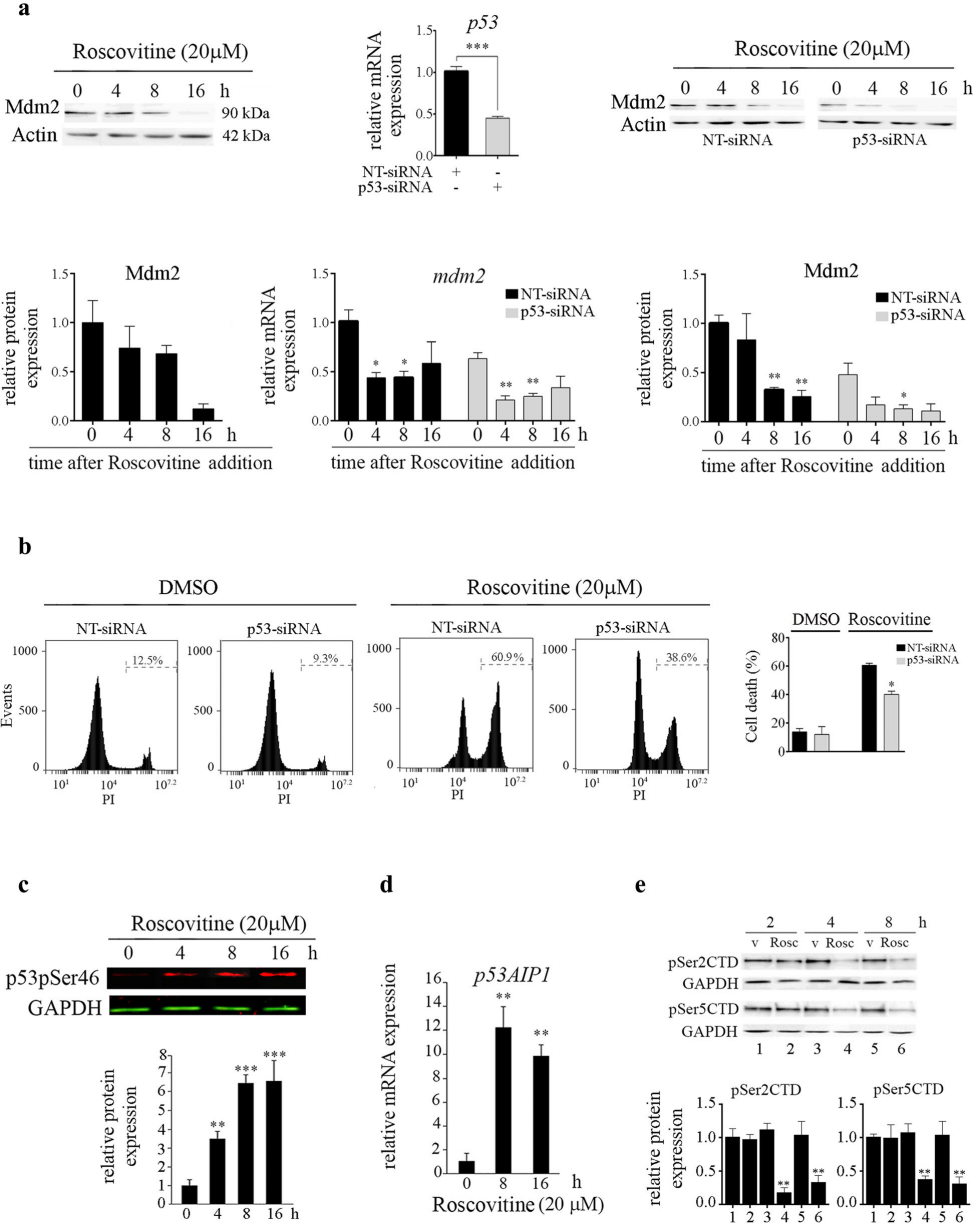
Effect of siRNA-mediated downregulation of p53 in ROSC-treated hESCs. **a** Representative Western blot in H9 cells of Mdm2 at different time points after ROSC addition. Bar graphs show densitometric quantification. Analysis of *p53* (upper panel) and *mdm2* (lower panel) mRNA expression levels by Real Time RT-PCR in non-targeting (NT) or p53-siRNA transfected hESCs in the presence or absence of ROSC. Representative Western blot in siRNA transfected H9 cells of Mdm2 at different time points after ROSC addition. Bar graphs show densitometric quantification. **b** H9 hESCs were transfected with NT-siRNA or p53-siRNA and exposed to ROSC (20 μM during 16 h). Representative histograms of PI-stained transfected cells left untreated or exposed to ROSC. Bar graphs show the percentage of cell death. Each bar represents the mean ± SD of three independent experiments. **c** Time course of p53 phosphorylation at serine 46 upon ROSC treatment was analyzed by Western blotting using anti-phospho-p53Ser46 (p53pSer46) specific antibody. GAPDH served as loading control. **d** Time-course analyses of p53Ser46 target *p53AIP1* mRNA expression levels by Real Time RT-PCR in ROSC-treated or untreated hESCs. *Rpl7* expression was used as normalizer. In all cases, a paired Student’s t test was used to test for significant differences between ROSC-treated and untreated samples *P < 0.05, **P < 0.01, ***P < 0.001. **e** Cells were treated with vehicle (v) (lanes 1–3-5) or 20 μM ROSC (lanes 2–4-6). At the indicated time points, cell lysates were analyzed by immunoblotting using the indicated antibodies. Bar graphs show densitometric quantification

To address whether the increase in nuclear p53 was accompanied by an increase in p53 transcriptional activity, the levels of four well characterized p53-responsive genes (Mdm2, p21^Cip1^, PUMA and PMAIP1/NOXA) were measured by quantitative RT-PCR in ROSC-treated and untreated hESCs [30]. As shown in Fig. 3c, a robust induction of *p21*^*Cip1*^ and, to a lesser extent, *puma* and *noxa* mRNAs expression levels were determined after 20 μM ROSC addition. Unexpectedly, we found that the levels of the well-known negative regulator of p53, *mdm2* transcript declined after treatment. The observed decrease in *mdm2* mRNA levels was reflected at the protein product as judged by Western blot analysis (Fig. 4a, left panel). In order to gain insight into the involvement of p53 in the regulation of Mdm2 gene in H9 cells we used siRNA-mediated gene silencing. We assessed the efficacy of the p53-trageting siRNA by real time RT-PCR in cells transfected with either non targeting control siRNA (NT-siRNA) or p53 specific siRNA. As shown in Fig. 4a, a significant decrease in p53 mRNA levels was observed in p53-siRNA transfectants. We found that siRNA-mediated downregulation of p53 led to a reduction of *mdm2* mRNA levels in hESCs. Additionally, we determined that the p53 targeting-siRNA cooperated with ROSC to further reduce Mdm2 levels (Fig. 4a). Importantly, these results suggest that in hESCs, ROSC selectively inhibits the expression of *mdm2*, which may lead to the observed stabilization and accumulation of p53. These findings are consistent with previous data showing that ROSC exposure decreased the rate of *mdm2* transcription [31]. As expected, cell death was reduced from 61.45 ± 1.85% in NT-siRNA transfected cells to 40.55 ± 2.95% in p53-siRNA transfected cells after ROSC treatment (Fig. 4b). Importantly, the fact that H9 cells with p53 mRNA levels reduced by approximately 50% exhibit a lower rate of cell death after ROSC exposure, suggests that p53 is a relevant player in the regulation of pluripotent cells apoptosis.

Post-translational modifications of specific residues contribute to p53 stabilization and are thought to determine the choice among the several p53-regulated programs [32]. Phosphorylation is also important for stabilization of p53 under certain stress conditions. Thus, in order to determine whether the nuclear accumulation of p53 in response to ROSC was accompanied by phosphorylation events, we performed immunofluorescence assays using a specific antibody against phospho-p53 serine15 (p53pSer15). In contrast to what we observed in camptothecin treated cells (1 μM for 1 h) (used as a positive control), the p53 proteins that accumulated in the nuclei after ROSC treatment (20 μM for 4 h) were not phosphorylated at the serine 15 site (Fig. 3b). This was previously described in human diploid fibroblasts exposed to ROSC [33]. Consistent with the lack of p53 phosphorylation at serine 15, no appreciable γH2AX immunoreactivity (a marker of DNA damage) was observed in ROSC treated cells. Conversely, a robust induction of γH2AX was observed in camptothecin treated H9 cells (Fig. 3b).

Phosphorylation of p53 at serine 46 is an important cell fate determining factor promoting p53-mediated transcriptional activation of pro-apoptotic genes [34]. To further investigate whether ROSC induces any other phosphorylation of p53 we performed Western blot analysis using antibodies against phospho-p53 serine 46 (p53pSer46). As shown in Fig. 4c, phosphorylation at this residue was detectable 4 h after ROSC addition resulting more evident at 16 h post treatment. Thus, although p53 accumulated in the nucleus after exposure either to camptothecin or ROSC, modification of p53 at serine 15 appears to be mainly associated with genotoxic stress rather than with CDK inhibition in this cell type. Besides, serine 46 phosphorylation is found to play a critical role in p53-mediated apoptotic genes induction such as p53-regulated Apoptosis-Inducing Protein 1 (p53AIP1) [34]. To this end, we examined whether *p53AIP1* mRNA levels were induced upon ROSC treatment. Real time quantitative RT-PCR analysis revealed a strong induction of *p53AIP1* transcripts from about 8 h onwards after ROSC exposure (Fig. 4d).

### ROSC modulates phosphorylation status of the CTD of the RNA polymerase II in hESCs

Assessment of the phosphorylation status of the CTD of the largest subunit of the RNA polymerase II, which is composed of 52 repeats of the consensus heptapeptide Tyr1- Ser2-Pro3-Thr4-Ser5-Pro6-Ser7, offers a functional assay to test CDK7 and CDK9 activities. CDK7 phosphorylates RNA pol II serine 5, whereas CDK9 phosphorylates predominantly serine 2 (though it can phosphorylate both under certain conditions in different cell types). Phosphorylation on these residues is of known importance to the regulation of transcription by CDK7 and CDK9 [35–37]. To investigate whether ROSC was affecting the activation of RNA pol II in H9 cells we analyzed the levels of RNA polymerase II phosphorylated at serine 2 and serine 5 by Western blotting. As shown in Fig. 4e, a significant time-dependent loss of phosphorylation at serine 2 and 5 residues was observed when hESCs were incubated with ROSC. These results suggest that, as occurs in several cell types [38], ROSC is inhibiting transcriptional CDKs in hESCs. These findings are further supported by the decline of short-lived mRNAs such as *mdm2* and *mcl-1*. Worth noting, we observed that even though ROSC exposure markedly reduced the phosphorylation of serine 2 and 5 within the CTD of RNA Pol II, the presence of this CDK inhibitor led to a significant increase in the levels of *p21*^*Cip1*^, *puma*, *noxa* and *p53AIP1* mRNAs.

### Inhibition of CDK2 did not induce cell death in hESCs

**Fig. 5:**
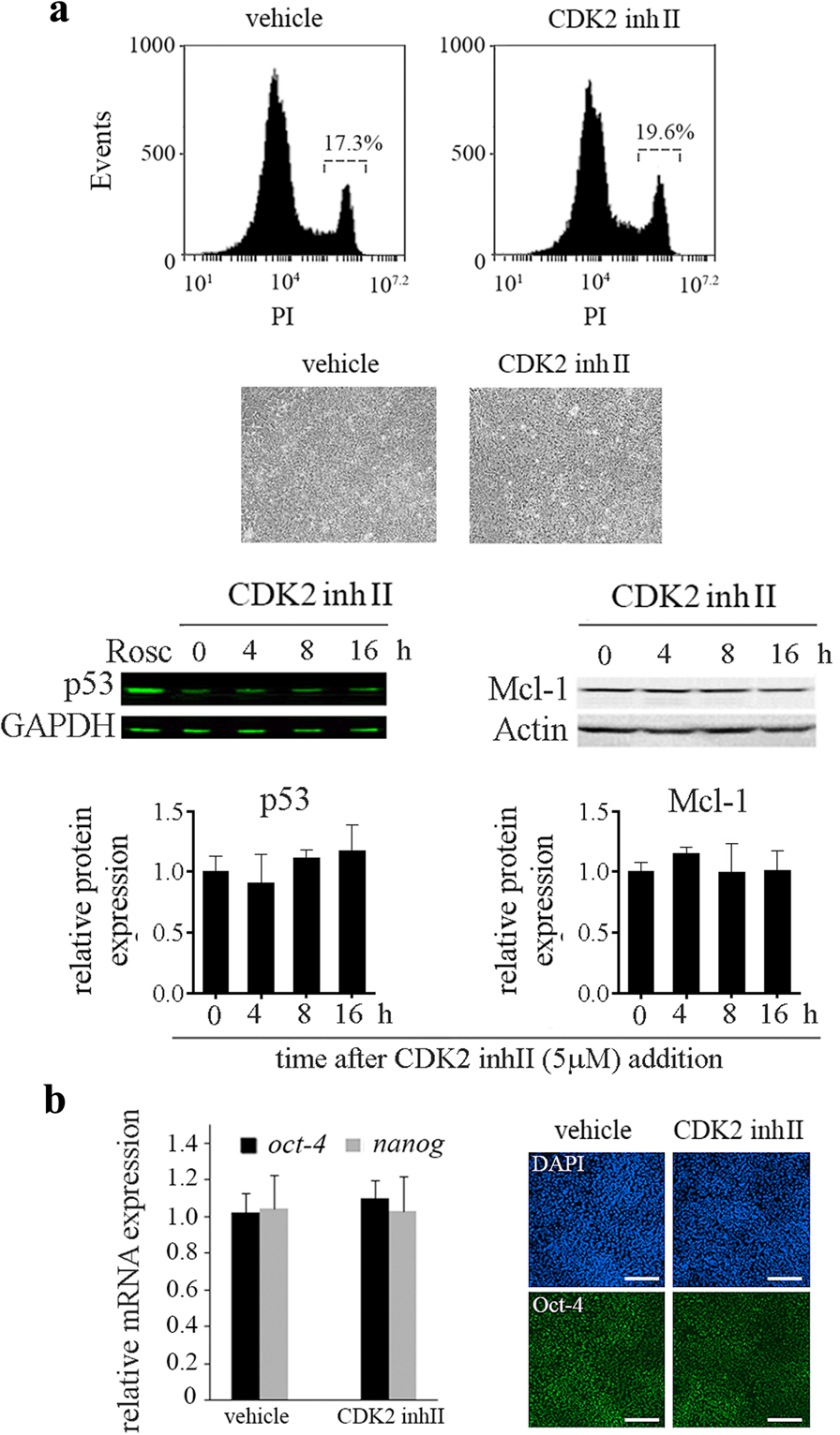
Small molecule inhibition of CDK2 did not induce cell death in hESCs. **a** Representative histograms of PI stained cells exposed or not to CDK2 inhibitor II (5 μM) for 24 h. Percentage of PI+ cells was determined by flow cytometric analysis (top panel). Representative photomicrographs of H9 colonies and HF treated or not with 5 μM CDK2 inhibitor II over a 24 h period (bottom left panel). p53 and Mcl-1protein levels were analyzed in the presence or absence of CDK2 inhibitor by Western blot. GAPDH and Actin were used as loading controls. Bar graphs show densitometric quantification (bottom right panel). **b** Pluripotency markers *oct-4* and *nanog* mRNA expression levels by Real Time RT-PCR in CDK2 inhibitor II-treated or untreated (vehicle) hESCs. *Rpl7* expression was used as normalizer (right panel). The mean ± SEM from three independent experiments are shown. In all cases, a paired Student’s t test was used to test for significant differences between ROSC-treated and untreated samples. *P < 0.05. Immunofluorescence photomicrographs of CDK2 inhibitor II-treated H9 cells (5 μM during 72 h). Representative images of H9 cells stained with primary antibody against Oct-4. Nuclei were counterstained with DAPI. Scale bars represent 100 μm

We then asked whether inhibition of CDK2 would result in similar effects as those seen after ROSC treatment in hESCs. To address this question, we exposed pluripotent cells to 5 μM CDK2 inhibitor II for 24 h. To confirm the effectiveness of this small-molecule inhibitor we performed Western blot analysis to evaluate cyclin E abundance in CDK2 inhibitor II-treated H9 cell, as it has been shown that raised cyclin E levels are a reflection of inactive CDK2 [39]. Scanning densitometries of immunoblots revealed a significant increase in cyclin E protein levels in H9 cells exposed to this CDK2 inhibitor when compared to that determined in untreated counterparts. This result has been previously reported by our group [40]. Importantly, when CDK2 activity was impaired no considerable changes in hESCs viability and morphology were observed (Fig. 5a). Moreover, the protein levels of p53 and Mcl-1 did not vary significantly upon CDK2 inhibition (Fig. 5a) and no alterations on cell cycle distribution were detected (data not shown). These findings are in accordance with previous studies describing that knockdown of CDK2 in mouse ESC had little effect on cell cycle and did not significantly affect their viability [17]. Then, we assessed the ability of this CDK2 inhibitor to influence the expression of pluripotency markers in hESCs. As illustrated in Fig. 5b, no appreciable changes in the expression levels of Oct-4 and Nanog were observed after a 72 h exposure.

## Discussion

CDKs, are important regulators of the cell cycle, have well-defined roles in transcription regulation and debatable roles in apoptosis. Currently, the role of CDKs in regulating hESCs proliferation and viability has not been fully addressed. For this reason, in the present study we aim to investigate the effects triggered by ROSC, a well-known CDK inhibitor, in hESCs.

Herein, we demonstrated that hESCs are sensitive to the anti-proliferative effects of ROSC. One of the most prominent effects of ROSC is the inhibition of CDK2 and CDK1, even though, many studies have provided evidence that the anti-proliferative effects of ROSC are produced by its ability to target several CDKs rather than only one of them [15].

Suppressed transcription and decreased phosphorylation of RNA pol II were documented in cells treated not only with ROSC but also with other CDK inhibitors [27, 41]. In this regard, we observed a marked reduction in the phosphorylation levels of serine 2 and serine5 residues (targets of transcriptional CDKs) within the CTD of RNA pol II upon ROSC treatment. Labile transcripts, as those with short half-lives encoding cell cycle and apoptosis regulators, are most sensitive to CDK-mediated transcriptional inhibition as their levels rapidly decrease upon transcription inhibition. In this regard, we determined a marked decrease in *cyclin E, A2, B1, B2* and *cdk1* mRNA expression levels in hESCs exposed to ROSC, presumably due to its ability to affect CDK7/9-dependent transcription. In this context, it is conceivably that the observed alterations in the expression levels of key cell cycle regulators may significantly contribute to the cell cycle arrest induced by CDK inhibition.

Importantly, we also demonstrated that ROSC triggers apoptosis in hESCs but not in primary fibroblasts, and found that the apoptotic process encompasses caspase-9 and -3 activation and PARP cleavage. As occurred in several cell types [42] we found that ROSC led to Mcl-1 down-regulation in hESCs and FH. Mcl-1 is an important transcriptional target of RNA pol II and a short-lived transcript, therefore repression of transcription may be the cause of its relatively rapid elimination. The fact that Mcl-1 levels can be both rapidly induced and lost places it as an ideal candidate to detect and respond to survival or death signals. The observation that ROSC induced cell death in H9 cells but not in FH suggests that these pluripotent cells rely more heavily on Mcl-1 for their survival than FH. In line with this finding, Huskey et al. reported that siRNA knockdown of *mcl-1* induces cell death in hESCs but not in human embryonic kidney cells (HEK293) [17]. In comparison to other BH3-only proteins, NOXA shows the most restricted potential to sequester anti-apoptotic factors as it preferentially binds to Mcl-1 [43]. Therefore, susceptibility to NOXA-induced cell death mainly relies on Mcl-1 activity relative to other pro-survival Bcl-2 family members [44]. It stands to reason that in ROSC-treated hESCs the observed down-regulation of Mcl-1, which was accompanied by up-regulation of *noxa*, may allow a transient increase in free NOXA concentration. Thus, we suspect this factor may play an important role in ROSC-induced cell death.

Several drugs activate p53 function by partial inhibition of RNA polymerase II-mediated transcription [45–47]. This may be attributed, at least in part, to *mdm2* down-regulation, another short-lived transcript that encodes an E3-ubiquitin ligase negatively regulating p53 protein level. In this sense, we found that in H9 cells ROSC treatment led to p53 stabilization and nuclear localization, which coincided with decreased levels of Mdm2. This phenomenon has been previously observed in different cell lines [31]. We also found that in ROSC-treated hESCs p53 underwent site specific phosphorylation at serine 4*6*. In line with previous reports, this post-transcriptional modification was concomitant with a robust induction of *p53AIP1* gene expression, a well-known transcriptional target of p53pSer46 [34, 48].

Earlier reports provide evidence that p53 is able to activate select target genes and trigger an apoptotic program when mRNA synthesis is inhibited; it thus appears that in hESCs this transcription-inhibitory effect also varies among p53-regulated promoters, at least at the concentration of ROSC used in our experiments. In fact, we observed that while some p53 target genes including *p21*^*Cip1*^, *puma, noxa* and *p53AIP1* were clearly up-regulated in ROSC-treated cells, other p53 transcriptional targets such as *mdm2* were downregulated. These results suggest that, as previously described for cancer cells [46, 47], in pluripotent cells a specific subset of p53 target genes may bypass the requirement for CDK9 activity and/or RNAP II complete phosphorylation when mRNA synthesis is broadly comprised. It thus appears that hESCs may be endowed with a p53-mediated safeguard mechanism to confront dysfunctions in transcription.

To further elucidate the consequence of CDK inhibition in H9 cells, we used a small molecule CDK2 inhibitor and found that impairment of CDK2 activity did not have considerable effects on cell proliferation nor in cell death. Our results are consistent with previous findings describing that the loss of CDK2 can be compensated by CDK1, which has been proved to be capable of substituting the missing activity of any other CDK [10, 49]. Further supporting these findings, Huskey et al. recently described that depletion of cyclin D1, E1, E2, or CDK2 did not have considerable effects on mouse ESC proliferation and did not induce cell death. Nevertheless, in the same study researchers found that CDK1 inhibition significantly increased cell death in mouse ESCs and in two independently derived hESC lines. Concerning to CDK1 inhibition, Canduri et al found, after analyzing the number of hydrogen bonds between ROSC-CDK1 and ROSC-CDK2, that the ROSC-CDK1 complex presents a higher number of intermolecular hydrogen bonds, indicating that this purine analogue has higher affinity for CDK1 than for CDK2 [50]. According to the aforementioned property of ROSC, it is conceivable to hypothesize that the vast majority of the effects triggered by this CDK inhibitor in hESCs may be due to the impairment of CDK1 and transcriptional CDKs activities rather than a cause of CDK2 inhibition. It is also worth mentioning that our results are in stark contrast to those reported by Neganova et al., describing that siRNA-mediated silencing of CDK2 led to changes in H9 cells morphology, cell cycle profile, expression of pluripotency markers and CKIs [10]. In their study, they showed that down-regulation of key pluripotency markers such as Nanog and Oct-4 resumed upon restoration of CDK2 expression levels (4 days after transfection and onwards) suggesting a “reversible” onset of differentiation. Thus, it seems that the absence of CDK2 was not compensated by others CDKs in H9 cells, as the majority of the cells (96.9%) were arrested at G1 phase after CDK2-siRNA transfection [10].

## Conclusion

hESCs, a promising cell source for research and clinical applications, are very prone to undergo apoptosis; however, the mechanisms underlying this proneness are not fully understood. Herein, we demonstrate that combined inhibition of cell cycle-associated and transcriptional CDKs by ROSC triggers programmed cell death in hESCs but not in somatic cells (FH). The apoptotic process involves caspase-9 and caspase-3 activation, PARP cleavage, p53 stabilization and site specific phosphorylation at serine 46, transcriptional induction of *p53AIP1* and repression of Mcl-1 concomitant with *noxa* and *puma* up-regulation. Furthermore, we determined that the role of CDK2 inhibition seems to be at best accessory, as an active CDK2 was not required to ensure hESCs survival. Our findings broaden the insights into hESCs apoptosis and may be helpful for establishing pro-survival strategies for its use in therapeutic purposes.

## Methods

### Cell culture

The hESC line, WA09 (H9), was obtained from WiCell Research Institute at low passages. H9 cells line were expanded on an inactivated mouse embryonic fibroblast feeder layer in medium comprised of Dulbecco’s Modified Eagle’s Medium/Ham’s F12 (DMEM/F12) supplemented with 20% knockout serum replacement (KSR), 2 mM L-glutamine, 2 mM non-essential amino acids, 50 μg/ml streptomycin, 100 U/ml penicillin, 0.1 mMβ-mercaptoethanol and 4 ng/ml of basic fibroblast growth factor. All these reagents were obtained from Thermo (Carlsbad, CA, USA). H9 cells were incubated with 1 mg/ml collagenase IV (Thermo, CA, USA) and seeded into diluted (1/40) Matrigel (Corning) -coated dishes containing inactivated mouse embryonic fibroblast conditioned medium. hESCs were routinely screened for *Mycoplasma* sp. contamination as previously described [51].

Human fibroblasts (HF) were prepared as primary cultures from freshly obtained foreskins after surgery. Surgically discarded tissue was cropped into strips using a sterile scalpel. Strips were subjected to an overnight digestion with dispase (Invitrogen, CA, USA) and then followed by careful removal of the epidermis. The remaining dermis was placed in high glucose DMEM, 10% FBS (vol/vol). Within 8–10 days outgrowths of fibroblasts appeared.

### Drug treatments

Cells were seeded 24 h before incubation with ROSC (Sigma St. Louis, MO, USA). Proteosome inhibitor MG-132 (474,790, CAS 133407–82-6, Calbiochem, CA, USA) and CDK2 inhibitor II (sc-221,409, Santa Cruz Biotechnology, CA, USA) were also used. Inhibitors were added to cell cultures such that the final DMSO concentrations were kept constant at 0.25% (v/v). Cells treated with 0.25% DMSO were always included as controls.

### Cell viability assay

Cells were seeded in 48-well plates at a density of 3 × 10^4^ cells per well. After 16 h of treatment, 50 μg/well of activated 2,3-bis (2-methoxy-4-nitro-5-sulfophenyl)-5 [(phenylamino) carbonyl]-2 H-tetrazolium hydroxide (XTT) in PBS containing 0.3 μg/well of N-methyl dibenzopyrazine methyl sulfate (PMS) were added (final volume 100 μl) and incubated in a humidified atmosphere for 4 h at 37 °C. Metabolic activity was measured using a spectrophotometer at 450 nm and subtracting the background absorbance at 690 nm.

### Trypan blue staining

Cells were plated in 6-well plates at a density of 1 × 10^5^ cells/ml. After treatment, 10 μl of the cell suspension (adherent and detached cells) were transferred to a 1.5 ml tube, stained for 5 min at room temperature with 0.4% Trypan blue (Sigma, St. Louis, MO, USA) in 0.85% saline solution and the mixture incubated for 2 min. Cells stained blue and unstained were counted in a Neubauer hemocytometer.

### Immunostaining and fluorescence microscopy

Cells were rinsed with ice-cold PBS and fixed in PBSA (PBS with 0.1% bovine serum albumin) with 4% formaldehyde for 45 min. Cells were washed two times with PBS and permeabilized with 0.1% Triton X-100 in PBSA with 10% normal calf serum for 30 min, washed twice and stained with the corresponding primary antibodies. Then, fluorescent dye–labeled secondary antibody along with DAPI were added and cells were incubated for 45 min at room temperature protected from light. H9 cells were examined under a Nikon Eclipse TE2000-S and images were acquired with a Nikon DXN1200F digital camera. The following primary antibodies were use: α-γH2AX (ab2893), α-p53 (DO-1) (ab1101) (Abcam Inc., Cambridge, MA, USA) and α-phospho-p53serine15 (cat.9284) (Cell Signaling Technology, Beverly, MA, USA).

### Flow cytometric analysis of cell cycle distribution

For DNA content analysis, cells were fixed in 70% ethanol, rehydrated in PBS, and treated for 30 min with RNase A (100 μg/ml) and for 5 min with Propidium Iodide (PI) (1 mg/ml). Fluorescence intensity was determined by flow cytometry on a BD Accuri C6 flow cytometer (BD Biosciences, San Jose, CA). The percentage of cells in each stage of the cell cycle was assessed by the FlowJo v10.0.7’s univariate platform (https://www.flowjo.com /solutions/flowjo/download).

### Flow cytometric analysis of bromodeoxyuridine (BrdU) incorporation and cell cycle distribution

The distribution of cell populations throughout the cell cycle and the fraction of cells capable of incorporating BrdU was determined using the BrdU Flow Kit (BD Biosciences, San Jose, CA, USA). After the corresponding treatment, cells were incubated with BrdU (10 μM) for 30 min. Cultures were processed as per manufacturer’s instructions. Fluorescence intensity was quantified on a BD Accuri C6 flow cytometer. Data were analyzed using BD AccuriC6 software.

### Flow cytometric analysis of cell viability using PI

After 16 h of ROSC treatment single-cell suspensions were obtained by treatment with accutase (37 °C for 5–10 min). Cells were then centrifuged at 200 x g for 5 min and resuspended up to 10^6^ cells/ml in FACS Buffer (2.5 mMCaCl_2_, 140 mM NaCl and 10 mM HEPES, pH 7.4). Next, 100 μl of cellular suspension were incubated with 5 μl of PI (50 μg/ml) in PBS for 5 min protected from light. Finally, 400 μl of FACS Buffer were added to each tube and cells were analyzed by flow cytometry. Results were expressed as the percentage of cells that displayed PI fluorescence (non-viable) to the total number of cells processed. Fluorescence intensity was determined on a BD Accuri C6 flow cytometer. Data were analyzed using BD AccuriC6 software.

### Reverse transcription polymerase chain reaction and real time PCR

Total RNA was extracted using TRIzol reagent (Thermo, Carlsbad, CA, USA) as per manufacturer’s instructions. cDNA was synthesized using MMLV reverse transcriptase (Promega, Madison, WI, USA) from 500 ng of total RNA. Quantitative PCR studies were conducted using SYBR® Green-ER™ qPCRSuperMix UDG (Thermo, Carlsbad, CA, USA).

Primers used were the following: Cyclin A2 forward 5′-CCTGCAAACTGCAAAGTTGA-3′, reverse 5′-AAAGGC AGCTCCAGCAATAA-3′; Cyclin B1 forward 5′-CAAGCCCAATGGAAACATCT-3′, reverse 5′-GGATCAGCTCCATCTTCTGC-3′, Cyclin B2 forward 5′-ACTGCTCTGCTCTTGGCTTC-3′, reverse 5′- TTTCTCGGATTTGGGAACTG-3′; Cyclin D1 forward 5′-GATCAAGTGTGACCCGGACT-3′, reverse 5′-TCCTCCTCCTCTTCCTCCTC-3′; Cyclin D2 forward 5′-TTGTTCCCGAGCGATAGATG-3′, reverse 5′- ACCAGAAGCGAAGAGTAACC-3′; Cyclin E forward 5′-AGGGGACTTAAACGCCACTT-3′, reverse 5′-AGGGGACTTAAACGCCATT-3′; CDK1 forward 5′-GCTGGCTCTTGGAAATTG-3′, reverse 5′-GTTAGTCAATGGGTATGGTAG-3′; CDK2 forward 5′-CCCTTTCTTCCAGGATGTGA-3′, reverse 5′-TGAGTCCAAATAGCCCAAGG-3′; CDK4 forward 5′-TGCAACACCTGTGGACATGTG-3′, reverse 5-´ ATTTGCCCAACTGGTCGG-3′; CDK6 forward 5′-TCCCTCCTTTGAAGTGGATG-3′, reverse 5′-GTCACCTGGGGCTAAATGAA-3′; CDK7 forward 5′-GTCTCGGGCAAAGCGTTATG-3′, reverse 5′-TTGGTTGGTGTTCTTATCTCTGG-3′; CDK9 forward 5′-CACCAACTCGCCCTCATC-3′, reverse 5′-GCCTGTCCTTCACCTTCC-3′; Mcl-1 forward 5′-GGGCAGGATTGTGACTCTCATT-3′, reverse 5′-GATGCAGCTTTCTTGGTTTATGG-3′; Bcl-xL, forward 5′-TGCGTGGAAAGCGTAGACAAG-3′, reverse 5′-GTGGGAGGGTAGAGTGGATGG-3′; p21^Cip1^forward 5′-CCGAAGTCAGTTCCTTGTGG-3′, reverse 5′-GGA TTAGGGCTTCCTCTTGG-3′; Mdm2 forward 5′-GAATCATCGACTCAGGTACATC-3′, reverse 5′-TCTGTCTCACTAATTGCTCTCCT-3; RPL7 forward 5′-.AATGGCGAGGATGGCAAG-3′; reverse 5′-TGACGAAGGCGAAGAAGC-3′; Puma, forward 5′-GACCTCAACGCACAGTACGAG-3′, reverse 5′-AGGAGTCCCATGATGAGATTGT-3′; NOXA, forward 5′-ACCAAGCCGGATTTGCGATT-3′, reverse 5′-ACTTGCACTTGTTCCTCGTGG-3′; p53AIP1, forward 5′-CACCCACCCGTTGCCTTC-3′, reverse 5′-GCTGAGTTCCTGCCTGTCC-3′. All samples were analyzed using an StepOnePlus Real Time PCR System (Applied Biosystems, Foster City, CA, USA) and were normalized to RPL7 gene expression.

### Western blotting

Cells were lysed in ice-cold radio-immunoprecipitation assay buffer supplemented with a protease and phosphatase inhibitor mixture, and protein concentration was determined using Bicinchoninic Acid Protein Assay (Pierce™, Rockford, IL, USA). Equal quantities of protein were subjected to 12% SDS-PAGE, and transferred to PVDF-FL membrane (Millipore, Billerica, MA, USA). The blot was blocked for 1 h in Odyssey blocking buffer (LI-COR Biosciences, Lincoln, NE, USA) containing 0.1% Tween 20 and incubated overnight at 4 *°*C in Odyssey blocking buffer, 0.05% Tween 20 and the corresponding primary antibodies. The blot was washed 4 *×* 5 min with Tris-buffered saline (TBS), 20 mM Tris-HCl, pH 7.5, 500 mM NaCl) containing 0.1% Tween 20 (TTBS), incubated for 1 h in Odyssey blocking buffer, 0.2% Tween 20 and IR-Dye labeled secondary antibodies (1:20.000, LI-COR Biosciences, Lincoln, NE, USA) and washed 4 × 5 min in TTBS, 1 *×* 5 min in TBS. The blot was scanned using the 680 nm and 780 nm channels at a scanning intensity of 4. Antigen-antibody complexes were visualized using the Odyssey Infrared Imaging System (LI-COR). The following primary antibodies were used: α-Actin (sc-1616), α-Bax (N-20) (sc-493), α-BclX_S_/_L_ (S-18) (sc-634), α-Mcl-1(S-19) (sc-819), α-CDK2 (M-2) (sc-163), α-CDK4 (H22) (sc-601), α-CDK6 (C-21) (sc-177) (Santa Cruz Biotechnology), α-phospho-p53serine46 (cat.2521) (Cell Signaling Technology, Beverly, MA, USA), α-p53 (DO-1) (ab1101) (Abcam Inc., Cambridge, MA, USA). Antigen/primary antibody complexes were detected with near infrared-fluorescence-labeled, IR-Dye 800CW or IR-Dye 680RD, secondary antibodies (LI-COR Biosciences, Lincoln, NE, USA). In some cases, after electrophoresis proteins were transferred to a PVDF membrane (Bio-Rad, Hercules, CA, USA). Blots were blocked 1 h at room temperature in TTBS containing low-fat dry milk (5%). Membranes were incubated with the corresponding primary antibodies at 4 °C for 12 h in blocking buffer (3% low-fat dry milk in TTBS). Immunocomplexes were detected using the corresponding horseradish peroxidase-conjugated secondary antibodies and enhanced chemiluminescence reagents (SuperSignal West FemtoSystem, Thermo Scientific, Rockford, IL, USA). The following primary antibodies were used: α-PARP (sc-8007), α-Mdm2 (sc-965) and α-Actin (sc-1616) (Santa Cruz Biotechnology, Santa Cruz, CA, USA), α-active Caspase-3 (ab13847) (Abcam Inc., Cambridge, MA, USA), α-caspase-9 (cat. 9502) and α-Mcl-1 (cat.94296) (Cell Signaling Technology, Beverly, MA, USA) and α-phospho-RNA Polymerase II CTD Ser2 and Ser5 were a generous gift from Dr. A Kornblihtt, School of Sciences, University of Buenos Aires, Argentina. The following secondary antibodies were used: a horseradish peroxidase-conjugated α-rabbit IgG; α-mouse IgG or α-goat IgG. Densitometric quantification of immunoreactive bands was done by normalizing protein levels to that of the respective loading control, and the signal recorded for control cells was arbitrarily set to 1. Data shown are representative of three separate studies and expressed as means ± SD.

### Cell transfection and RNA interference

H9 cells were transfected with the corresponding siRNA using Lipofectamine RNAiMAX Transfection Reagent (Invitrogen, Carlsbad, CA) as per the manufacturer’s specifications. Briefly, 2 × 10^5^ cells/well (six-well plate) were transfected with Silencer Select Negative Control #2 (Ambion, cat. 4,390,846) or TP53 Silencer® siRNA (Ambion, siRNA ID: 106141). Twenty-four hours after transfection cells were treated according to requirements. Knockdown efficacy was analyzed by real time RT-PCR.

### Statistical evaluation

All of the results are expressed as the mean ± SEM. The student’s paired *t* test was used to determine significant differences between means, and *P* values below 0.05 were considered to be statistically significant.

## Data Availability

Data sharing is not applicable to this article as no datasets were generated or analysed during the current study.

## Abbreviations

CDK: Cyclin dependent kinase
CTD: Carboxy-terminal domain
hESCs: human embryonic stem cells.
